# Hemorrhagic Cholecystitis Due to Rupture of Underlying Anomalous Duplicated Cystic Arteries

**DOI:** 10.14309/crj.0000000000001503

**Published:** 2024-10-11

**Authors:** Victoria Diaz, George Trad, Sue Boo

**Affiliations:** 1 Department of Internal Medicine, Sunrise Health GME Consortium, Las Vegas, NV; 2 Southern Hills Hospital & Medical Center GME, Las Vegas, NV; 3 Department of Radiology, Sunrise Health GME Consortium, Las Vegas, NV

**Keywords:** hemorrhagic cholecystitis, cholecystitis, hemobilia, cystic artery

## Abstract

Hemorrhagic cholecystitis (HC) is a rare complication that can become rapidly fatal. Patients may present with hematemesis or melena, in addition to other common symptoms of acute cholecystitis. Delay in diagnosing HC postpones early intervention, and patients can quickly decompensate. We present a 33-year-old man with hematemesis and downtrending hemoglobin. Imaging revealed underlying anomalies of duplicate cystic arteries that ruptured, an occurrence never reported in the literature before. Bilateral cystic arteries were embolized successfully. This case demonstrates the importance of early consideration of HC as a differential. Recognition and timely diagnosis prompt urgent intervention, which can reduce morbidity.

## INTRODUCTION

Acute cholecystitis can transform into hemorrhagic cholecystitis (HC) when inflammation erodes nearby vasculature causing hemobilia, an extremely rare complication. Presenting with nonspecific symptoms that mimic the presentations of more common diagnoses, HC could easily be overlooked, delaying intervention. We present a case of HC in a young man with abdominal pain and hematemesis, subsequently found to be caused by the rupture of anomalous duplicate cystic arteries.

## CASE REPORT

**Figure 1:**
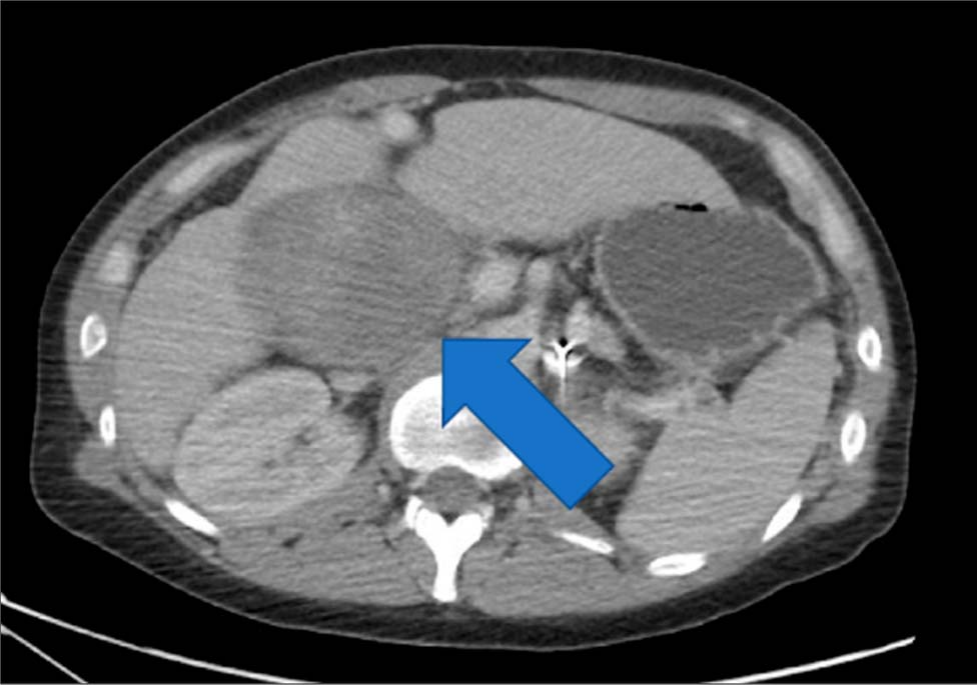
Computed tomography (CT) with intravenous contrast of the abdomen in the axial view showing biliary sludge and gallbladder distension with surrounding edematous changes at the blue arrow consistent with acute cholecystitis.

A 33-year-old man presented with complaints of worsening sharp right upper-quadrant pain and multiple episodes of hematemesis with associated syncopal episodes. The patient's medical history included decompensated alcohol-associated cirrhosis. Initial laboratory test result values revealed anemia, thrombocytopenia, elevated bilirubin, and aspartate aminotransferase. Initial computed tomography (CT) with intravenous contrast showed biliary sludge, cholelithiasis, and gallbladder distension (Figure 1). On admission, per Tokyo Guidelines for Cholecystitis 2018, the patient was deemed to have a definite diagnosis of acute cholecystitis, grade III (severe).^1^

**Figure 2:**
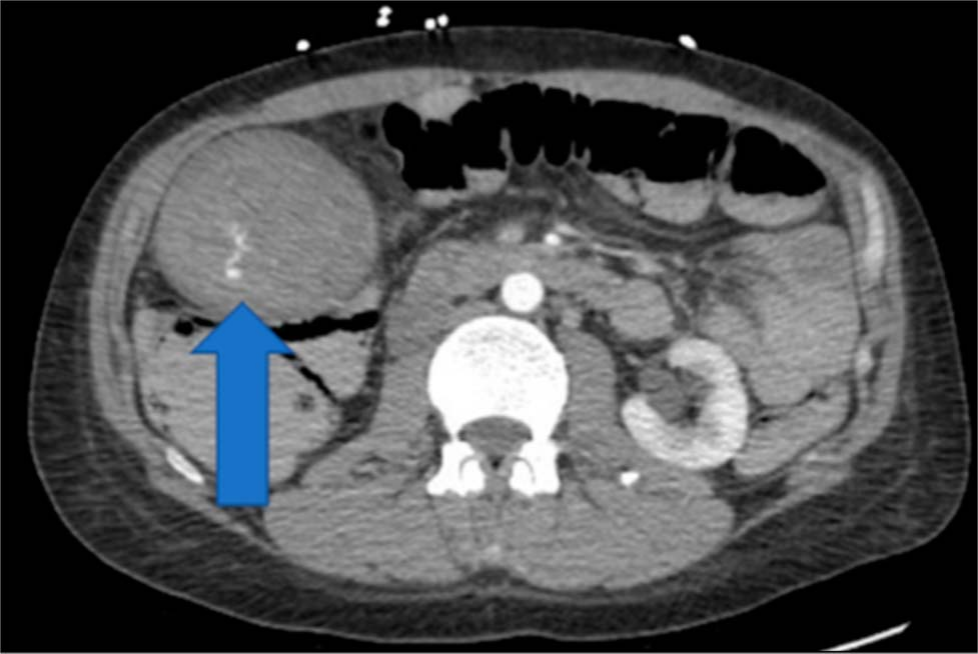
CT angiography (CTA) of the abdomen in axial view demonstrated a markedly distended gallbladder filled with a hematoma and small foci of arterial hemorrhages at the blue arrow.

**Figure 3:**
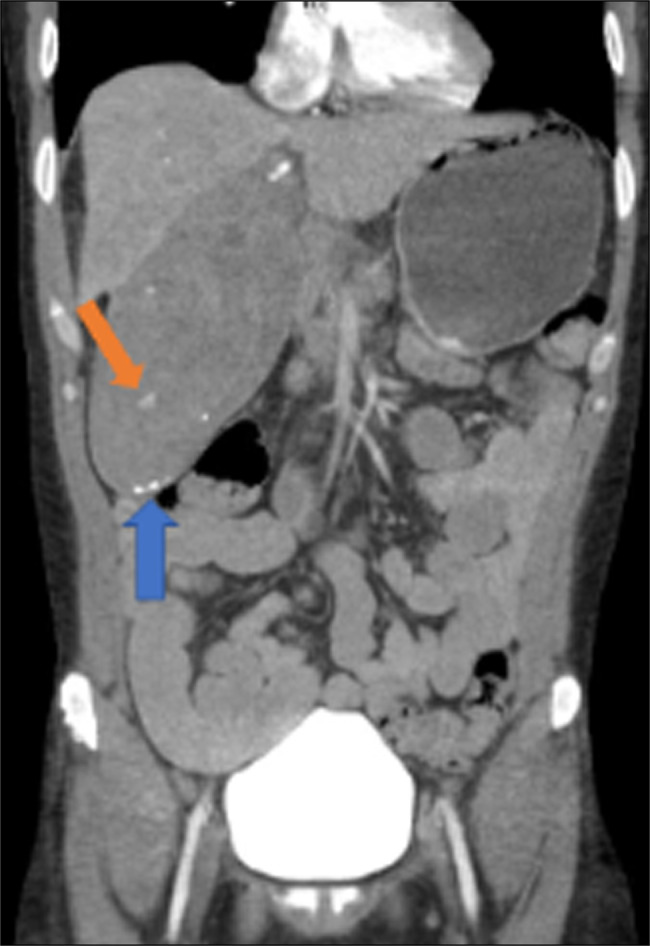
CTA in coronal view of the abdomen showed gallbladder filled with hematoma at the blue arrow and focus of hemorrhage at the orange arrow.

**Figure 4:**
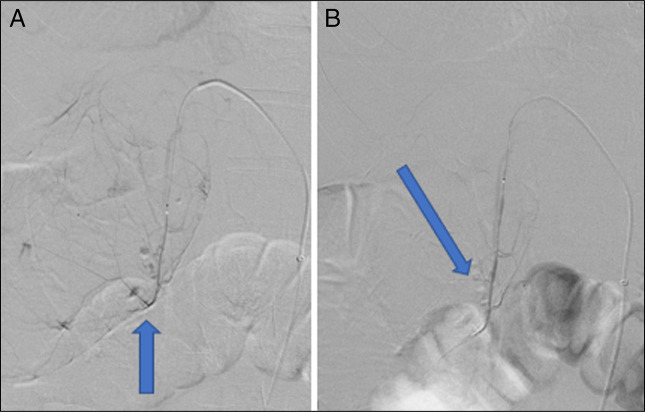
Selective digital subraction angiography demonstrates medial cystic artery with contrast extravasation at blue arrow (A) and successful gelfoam embolization at blue arrow by interventional radiology (B).

**Figure 5:**
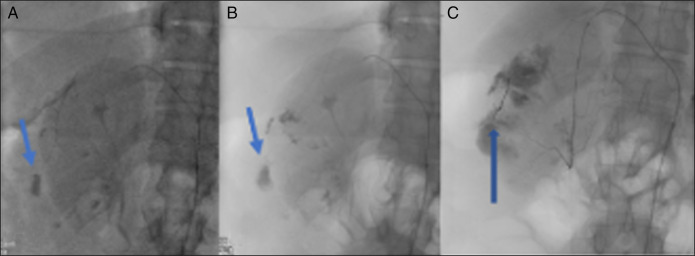
Selective fluoroscopy demonstrates lateral cystic artery hemorrhage at the blue arrows (A and B) with successful coil embolization at the blue arrow by interventional radiology (C).

Overnight, the patient's hemoglobin dropped from 9.2 to 5.3 GM/dL, prompting stat imaging. CT angiography findings included a markedly distended gallbladder filled with a hematoma and small foci of arterial hemorrhages (Figures 2 and 3). The patient was taken for selective visceral angiography of the right hepatic artery in an attempt to localize the source of the bleed, which revealed duplicate cystic arteries, each with contrast extravasation. Both arteries were embolized by interventional radiology with gel foam in the medial and coil in the lateral cystic arteries (Figures 4 and 5). A nuclear medicine bleeding scan with vascular flow demonstrated successful embolization. The patient was stabilized with blood transfusions, and his cirrhosis management was optimized. The patient was deemed a poor surgical candidate and was instructed to follow-up outpatient for possible cholecystectomy if his liver function improved.

## DISCUSSION

Although acute cholecystitis accounts for up to 10% of patients presenting with abdominal pain, very few cases will be complicated by HC, and even fewer secondary to cystic artery rupture.^1^ The incidence of HC is estimated to be only 0.55%.^2^ Hemobilia was first described as bleeding from the hepatobiliary system into the gastrointestinal (GI) tract in 1948.^3^ HC generally refers to acute cholecystitis complicated by intraluminal or peritoneal hemorrhage; however, it is not clearly defined in the literature because of its rarity.^4^ A patient presenting with HC because of rupture of anomalous duplicate cystic arteries has never been reported in the literature.

The leading causes of hemobilia are iatrogenic and trauma.^5,6^ Risk factors for the development of biliary hemorrhage include coagulopathies, platelet dysfunction, anticoagulation and/or antiplatelet therapy, long-term nonsteroidal anti-inflammatory drug use, chronic steroid treatment, malignancies, arteriovenous malformations, uremia, hepatobiliary or pancreatic inflammation, renal failure, and cirrhosis.^2,4,5,7^ Our cirrhotic patient had a greater propensity to bleed and higher risk of rapid decompensation. Patients on chronic anticoagulation who develop HC have an increased morbidity.^2,7^

HC and acute cholecystitis share similar pathophysiologies and are theorized to be caused by intraluminal pressure increases, most often because of obstruction decreasing blood flow, subsequently causing mucosal ischemia, necrosis, and erosion.^3,8,9^ This mechanism of pressure erosion likely led to the rupture of our patient's cystic arteries. Pressure increases can also cause cystic artery pseudoaneurysms to form and rupture, a more common cause of hemobilia; however, there was no evidence suggestive of pseudoaneurysm presence in our patient. In addition, cholelithiasis can cause microbleeding within the gallbladder, accounting for 9% of hemobilia cases.^10^

The symptomatic presentation of HC can be misleading because it can be nonspecific and have similar presentation to acute cholecystitis and GI bleeding.^11^ Jiang et al proposed that the diagnosis of HC be limited to patients presenting with both symptoms consistent with acute cholecystitis, as well as symptoms of hemorrhage and/or evidence of hemorrhage on imaging^4^. One-third of patients with hemobilia will present with a classic triad (Quincke triad) of symptoms described as biliary colic pain, icteric state, and GI bleeding, which can seem to be upper or lower in nature.^12^ Our patient's presentation was consistent with this triad, given his complaints of right upper-quadrant pain and hematemesis with jaundice noted on physical examination. Hemorrhagic biliary sludge or intraluminal gallbladder mass should also be considered as differentials.^13^

Ultrasound and CT are usually the initial imaging modalities used to investigate a suspected biliary source, but HC is a difficult diagnosis to make radiologically .^14^ CT has a sensitivity of 69.2% in detecting hemorrhage within the gallbladder, whereas ultrasound has only 38.4% sensitivity.^15^ With magnetic resonance imaging, differentiation can be made between the gallbladder lumen and hemorrhage at the wall and should be considered in the event that CT results are inconclusive or when trying to avoid radiation.^2,16^ Selective visceral angiography should be used when there is a high index of suspicion for HC because it can both confirm diagnosis, as well as accommodate therapeutic intervention.^5^

Management of HC prioritizes hemostasis and hemodynamic stabilization, followed by cholecystectomy, preferably laparoscopic.^13,17^ Localization of the affected vasculature can determine whether less-invasive angioembolization is a possibility.^4^ Unstable patients may require emergent exploratory laparotomy with open cholecystectomy to locate and control the culprit source, which is associated with poorer outcomes.^4^ One retrospective study emphasized that all 5 of the patients who required exploratory laparotomy and open cholecystectomy had initially presented in hemodynamically stable condition before quickly decompensating and found morbidity to be 21%.^4^ Alternatively, endoscopic retrograde cholangiopancreatography can be performed to remove the offending calculus and/or hematoma, relieving obstructive pressure and allowing bile secretion to resume. Once bile flow is restored, resolution of symptoms and laboratory abnormalities follow thereafter.^18^ Complications of HC include hemorrhagic shock, gallbladder perforation or necrosis, and hemoperitoneum.^5,15,19,20^ Because of its vague presentation, the risk of patients to quickly decompensate, and its potentially fatal nature, HC should always be considered as a differential to not delay intervention that could be life-saving.

## DISCLOSURES

Author contributions: V. Diaz: primary author organizing the conception/design of the work, drafting the work/reviewing it, making final approval of the version to be published, as well as agreement to be accountable for all aspects of the work in ensuring that questions related to the accuracy or integrity of any part of the work are appropriately investigated and resolved. G. Trad: initial provider to patient, responsible for all inpatient information and notes obtained, confirmation collection of patient consent, aiding in conception/design of case report, and editing of multiple drafts of paper. S. Boo: editing of multiple drafts of paper and coordination in obtaining imaging and discussions with radiology/interventional radiology. V. Diaz is the article guarantor.

Financial disclosure: None to report.

Previous presentation: This manuscript was developed from a previous abstract presented at the ACG Annual Meeting; October 22, 2023; Vancouver, BC, Canada.

Disclaimer: This research was supported (in whole or in part) by HCA Healthcare and/or an HCA Healthcare affiliated entity. The views expressed in this publication represent those of the author(s) and do not necessarily represent the official views of HCA Healthcare or any of its affiliated entities.

Informed consent was obtained for this case report.
